# SERS Detection of Hydrophobic Molecules: Thio-β-Cyclodextrin-Driven Rapid Self-Assembly of Uniform Silver Nanoparticle Monolayers and Analyte Trapping

**DOI:** 10.3390/bios15010052

**Published:** 2025-01-15

**Authors:** Qi Yuan, Yunqing Wang

**Affiliations:** 1 CAS Key Laboratory of Coastal Environmental Processes and Ecological Remediation, Yantai Institute of Coastal Zone Research, Chinese Academy of Sciences, Yantai 264003, China; 2 University of Chinese Academy of Sciences, Beijing 100049, China

**Keywords:** silver nanoparticle, β-CD-SH, self-assembly film, SERS, hydrophobic analyte

## Abstract

High-sensitivity and repeatable detection of hydrophobic molecules through the surface-enhanced Raman scattering (SERS) technique is a tough challenge because of their weak adsorption and non-uniform distribution on SERS substrates. In this research, we present a simple self-assembly protocol for monolayer SERS mediated by 6-deoxy-6-thio-β-cyclodextrin (β-CD-SH). This protocol allows for the rapid assembly of a compact silver nanoparticle (Ag NP) monolayer at the oil/water interface within 40 s, while entrapping analyte molecules within hotspots. The proposed method shows general applicability for detecting hydrophobic molecules, exemplified as Nile blue, Nile red, fluconazole, carbendazim, benz[a]anthracene, and bisphenol A. The detection limits range from 10^−6^to 10^−9^ M, and the relative standard deviations (RSDs) of signal intensity are less than 10%. Moreover, this method was used to investigate the release behaviors of a hydrophobic pollutant (Nile blue) adsorbed on the nanoplastic surface in the water environment. The results suggest that elevated temperatures, increased salinities, and the coexistence of fulvic acid promote the release of Nile blue. This simple and fast protocol overcomes the difficulties related to hotspot accessibility and detection repeatability for hydrophobic analytes, holding out extensive application prospects in environmental monitoring and chemical analysis.

## 1. Introduction

Surface-enhanced Raman scattering (SERS) is a highly sensitive spectroscopic technique that leverages the electromagnetic and chemical enhancement effects of metal nanostructures to significantly amplify molecular Raman scattering signals [1,2]. SERS has been extensively applied in chemistry and materials science, becoming an essential tool in molecular recognition, catalysis research, and biosensing [3,4,5]. The high sensitivity and rapid analysis capabilities have demonstrated exceptional advantages in trace substance detection across various fields [6,7,8].

The SERS substrate plays a crucial role in SERS detection. To achieve highly reproducible detection results, the microstructure of the substrate must exhibit a high degree of uniformity and repeatability [9,10]. In recent years, the preparation of SERS substrates via liquid/liquid self-assembly of noble metal nanoparticles (NPs) has gained significant attention due to its simplicity and convenience [11,12,13,14]. One application mode is to form a film at the liquid interface, and then transfer it to a solid surface. The test solution can be simply dropped onto or immersed in the SERS substrate for testing [15,16]. However, this mode suffers from drawbacks including the following: (1) The analyte molecules can hardly enter the “hot spot” between NPs, limiting the enhancement effect. (2) The coffee ring effect can lead to uneven distribution of analyte molecules on the SERS substrate, thereby affecting signal reproducibility and the accuracy of detection results.

In situ detection of compact NP monolayer films in liquids (at the oil/water interface or droplets onto a silicon wafer) represents another emerging application [17,18,19]. The key challenges of this strategy lie in (1) the prevention of uncontrollable aggregation of NPs at the interface due to the presence of salts in the solution and the resultant inhomogeneous substrate structure [20,21,22]; (2) ensuring that the analyte can effectively bind to the surfaces of NPs and be embedded in the hot spots during the film formation process. Benzyl butyl phthalate was reported to be directly embedded into the SERS-active nanogaps of the Au NP array, achieving a detection limit as low as 1.3 mg/kg [11]. Additionally, a droplet SERS detection method was proposed based on a nanocapillary pump model, which utilizes the reduction of Ag nanoparticle spacing during solvent evaporation to form hot spots, enabling ultratrace detection of various substances [23]. (3) The fast NP monolayer film formation speeds up detection efficiency. Most existing self-assembly film formation methods require a long time, longer than 20 min [24,25]. Song et al. [26] sped up the process by introducing 1H,1H,2H,2H-perfluorodecanethiol (PFT), but it occupies the metal NP surface and reduces the sensitivity of SERS detection.

β-cyclodextrin (β-CD) has successfully been applied in the field of SERS detection as a capture ligand due to its ability to encapsulate various guest molecules. Modifying the surface of SERS substrates with β-CD can utilize its unique cylindrical structure and the hydrophilic exterior with a hydrophobic interior to selectively capture analytes that are difficult to directly adsorb onto the surface of noble metals, such as hydrophobic or weakly charged molecules. For example, Guo et al. [27] achieved effective enrichment and significant Raman signal enhancement for multiple molecules through porous β-CD polymer/magnetic NPs. Zhang et al. [28] synthesized Au@Ag@β-CD NPs and mixed them with analytes to construct droplet-based SERS substrates, enabling sensitive detection of phthalates. Similarly, a monolayer film of Ag NPs was modified with mono-6-deoxy-6-thio-β-cyclodextrin (β-CD-SH) for methyl orange dye detection in water [15]. Despite these works having provided new insights for high-sensitivity detection, multiple steps of the SERS substrate preparation and additional surface modification are needed.

In this work, we developed a self-assembly protocol for monolayer SERS detection by simply mixing analyte, β-CD-SH and Ag NPs. With the aid of β-CD-SH, a Ag NP monolayer formed at the oil/water interface within 40 s, simultaneously the analyte molecules can be entrapped within hotspots. The proposed method shows general applicability for detecting hydrophobic molecules, exemplified as Nile blue, Nile red, fluconazole, carbendazim, benz[a]anthracene, and bisphenol A, with detection limits ranging from 10^−6^to 10^−9^ M, and the relative standard deviations of signal intensity being less than 10%. Moreover, this method was used to investigate the release behaviors of hydrophobic pollutant adsorbed on the nanoplastic surface in the water environment, suggesting that temperature, salinity, and dissolved organic matter affect the release of Nile blue from polymethyl methacrylate (PMMA) nanoplastic.

## 2. Materials and Methods

### 2.1. Reagents and Chemicals

Silver nitrate (AgNO_3_), sodium hydroxide (NaOH), sodium chloride (NaCl), hydrazine hydrate (HH), malachite green, potassium persulfate (KPS), hydrochloric acid (HCl), ethyl acetate, hexane, dichloromethane, and ethanol were purchased from Sinopharm Chemical Reagent Co., Ltd. (Shanghai, China). Nile red, fluconazole, bisphenol A, crystal violet, rhodamine 6G, polyvinyl pyrrolidone (PVP,) and 1H,1H,2H,2H-perfluorodecanethiol (PFT) were purchased from Shanghai Aladdin Biochemical Technology Co., Ltd. (Shanghai, China). Nile blue (NB), carbendazim, benz[a]anthracene, and methyl methacrylate (MMA) were purchased from Shanghai Macklin Biochemical Technology Co., Ltd. (Shanghai, China). The 6-deoxy-6-thio-β-cyclodextrin (β-CD-SH) was provided by Zhiyuan Biotechnology (Binzhou, China). Fulvic acid was purchased from Shanghai yuanye Bio-Technology Co., Ltd. (Shanghai, China).

### 2.2. Synthesis of Ag NPs

Silver NPs with an anionic ligand (Cl^−^) on their surface were synthesized according to previous work [29] as follows: 1.5 mL of NaOH (0.2 M), 1 mL of NaCl (0.01 M), and 0.5 mL of HH (0.1 M) were added to 7 mL of H_2_O and mixed thoroughly. Subsequently, 1 mL of AgNO_3_ (0.1 M) was added to 87 mL of deionized water, followed by the addition of the aforementioned mixed solution. The resulting mixture was stirred uniformly at room temperature for 30 min, and then the obtained Ag NPs were stored at 4 °C.

### 2.3. Ag NP Monolayer Film for SERS Substrate Preparation

2 mL of Ag NPs was concentrated to 1 mL by centrifugation and then introduced into a 5 mL glass beaker. Subsequently, 2 mL of a 10^−5^ M β-CD-SH solution (solvent composed of n-hexane and ethanol at a volume ratio of V_ethanol_/V_hexane_ = 2:1) was added to the beaker. After a static period of 40 s, a dense, lustrous silver film formed at the interface between the two phases. For the detection of hydrophobic analytes, the analytes were added to the β-CD-SH solution. For the detection of water-soluble analytes, they were introduced into the silver sol.

### 2.4. Nile Blue Release from Nanoplastic Under Varied Environmental Conditions

300 mg of PMMA nanoplastic was placed in a 10^−4^ M NB solution (15 mL), thoroughly mixed, and allowed to stand for 24 h to ensure complete adsorption of NB molecules. The mixture was then centrifuged and washed 10 times, after which the PMMA was stored at 4 °C for future use. Subsequently, 2 mg of the aforementioned PMMA was suspended in 10 mL of water and subjected to various environmental conditions (pH = 5, 7, 9; temperature: room temperature, 40 °C; salinity: 0.5 wt%, 1.5 wt%, 3.5 wt%; fulvic acid: 0.001 wt%, 0.005 wt%, 0.05 wt%, 0.5 wt%) for 12 h. The PMMA nanoplastics were then removed by centrifugation. Then, 2 mL of ethyl acetate was mixed with the aqueous solution, allowed to stand for phase separation, and then the NB molecules in the water were extracted. A 200 μL aliquot of the upper ethyl acetate layer was diluted 10-fold with β-CD-SH solution and assembled with Ag NPs to form a thin film. Raman spectroscopy was finally employed to quantitatively analyze the release of NB under the different conditions.

### 2.5. SERS Measurement

All Raman spectra were collected using a DXR Raman spectrometer; a 780 nm laser was focused onto the samples through a 50× microscope objective. Each measurement point had an exposure time of 5 s, with data collection repeated twice.

### 2.6. Instrumentation

Raman measurements were conducted using a DXR Raman microscope (Thermo Scientific, Norristown, PA, USA). Scanning electron microscope (SEM) images were obtained by a cold field emission scanning electron microscope (S4800, Hitachi, Tokyo, Japan).

## 3. Results and Discussion

### 3.1. β-CD-SH-Assisted Self-Assembly of Ag NPs into Monolayer Films

**Figure 1:**
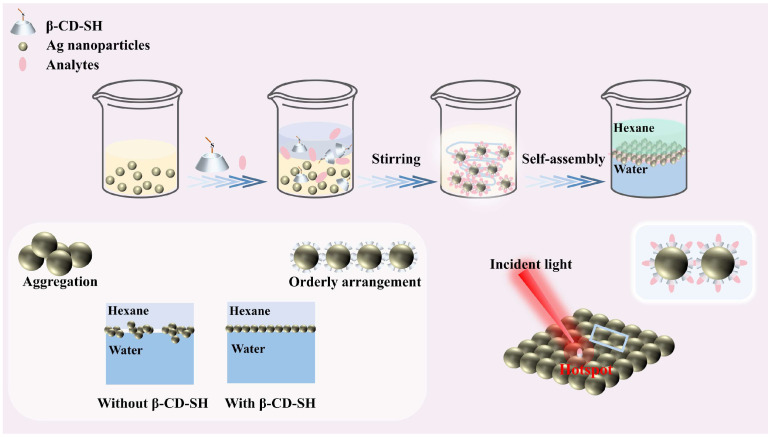
Schematic diagram of SERS detection utilizing the synergistic interaction between substrate self-assembly and target analyte capture.

A schematic illustration of the uniform formation of a Ag monolayer film under the assistance of β-CD-SH and the SERS detection process is depicted in Figure 1. After the addition of the organic phase to the Ag NP sol, the self-assembly process is initiated, simultaneously enabling the capture of test molecules. The addition of ethanol as a cosolvent is crucial to initiate the self-assembly process. However, it reduced retention of Cl^−^ on the surface of Ag NPs due to competitive adsorption and thus decreased electrostatic repulsion [30,31], causing aggregation of Ag NPs at the two-phase interface. This resulted in poor stability and uniformity of the assembled Ag monolayer film. The introduction of β-CD-SH into the system forms stable silver/sulfur bonds by means of its thiol groups with Ag NPs, while the rigid cavity structure at the other end maintains partial electrostatic repulsion, preventing the aggregation of Ag NPs [32,33]. Consequently, the assembled Ag NP film exhibits excellent uniformity and stability.

**Figure 2:**
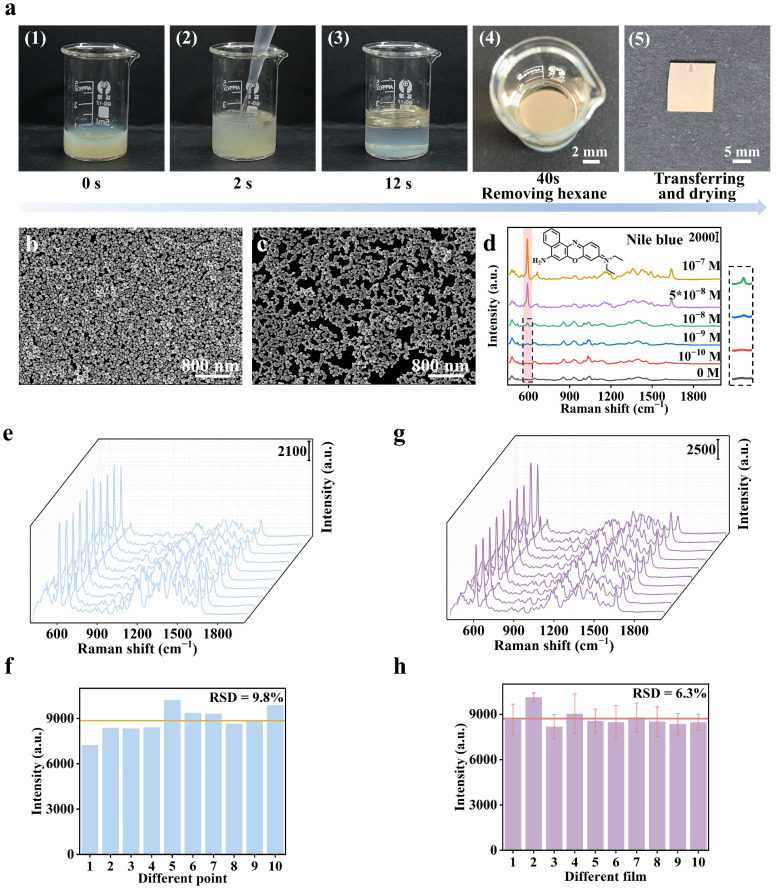
(**a**) Images of the self-assembly process of Ag NPs assisted by β-CD-SH, (**b**) SEM image of the film formed by Ag NPs with β-CD-SH modification, (**c**) SEM image of the film formed by Ag NPs without β-CD-SH modification, (**d**) SERS spectra of NB at different concentrations collected on a monolayer film (10^−5^ M β-CD-SH), (**e**) SERS spectra of 10^−7^ M NB collected from 10 random points on a monolayer film, (**f**) Intensity of the 593 cm^−1^ peak at 10 random points on the substrate, (**g**) SERS spectra of NB (10^−7^ M) detected on 10 batches of monolayer films prepared under the same conditions, and (**h**) Statistical distribution of SERS intensity at 593 cm^−1^ (NB, 10^−7^ M) corresponding to 10 batches of co-assembled films.

The entire self-assembly process requires only approximately 40 s and eliminates the need for tedious pretreatment and complex substrate preparation procedures (Figure 2a). Following the evaporation of hexane, the film can be rapidly transferred onto a silicon wafer surface. The optimal concentration of β-CD-SH is 10^−5^ M, at which Ag NPs are densely packed into a monolayer, forming a continuous macroscopic film (Figure 2b).

When the concentration of β-CD-SH is below 10^−5^ M, Ag NPs are prone to aggregation (Figure 2c), leading to an irregular and non-dense monolayer film which reduces the uniformity of the film, and the film at the interface exhibits a marked lack of metallic luster compared to those modified with β-CD-SH (Figure S1). An optimal condition for β-CD-SH is 10^−5^ M; SERS spectra of NB at various concentrations are shown in Figure 2d, and they can be clearly identified when the concentration is as low as 10^−9^ M. The signal intensity exhibits a good linear relationship with the NB concentration (Figure S2). It should be noted that the blank film shows a weak background signal of β-CD-SH (Figure S3), whereas it did not affect the identification of characteristic peaks of NB at 593 cm^−1^ attributed to C-C-C and C-N-C deformations [34,35]. When β-CD-SH concentration exceeds 10^−5^ M, a dense Ag film can still be formed, but the signal enhancement capability significantly decreases. As shown in Figure S4, peak intensity of NB (10^−7^ M) with 10^−5^ M β-CD-SH was 3.4 times and 5.15 times higher than that on films modified with 10^−3^ M and 10^−4^ M β-CD-SH, respectively. This is because excessive β-CD-SH occupies the Ag NP surface, and thus the portion of adsorbed host/guest complexes decreases, leading to a reduction in SERS signals [36]. In spiked experiments where a certain concentration of NB was added to the oil phase, the quantitative method demonstrated a recovery rate of 75% (Table S1).

Figure 2e presents the spectra of NB at 10^−7^ M collected randomly from 10 locations on the β-CD-SH-Ag film. The results reveal that the relative standard deviation (RSD) of the Raman intensity at 593 cm^−1^ is 9.8% (Figure 2f). Additionally, spectra randomly collected from 10 batches of Ag monolayer films also exhibited good consistency (Figure 2g), with an RSD of Raman intensity of 6.3% (Figure 2h).

### 3.2. Comparison with Typical SERS Film Strategies

**Figure 3:**
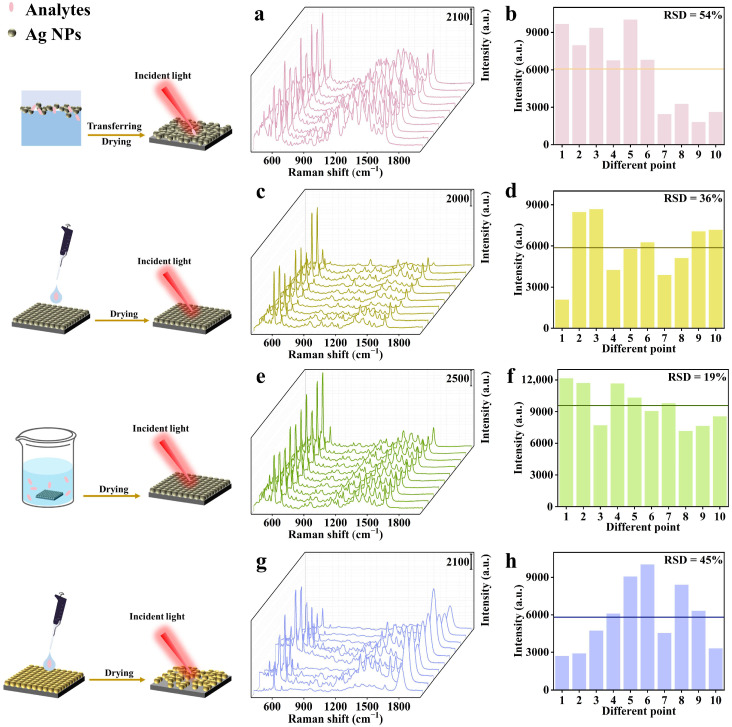
(**a**) SERS spectra of 10^−7^ M NB collected from 10 random points on a monolayer film (without β-CD-SH modification), (**b**) Intensity of the 593 cm^−1^ peak at 10 random points on the substrate (without β-CD-SH modification), (**c**) SERS spectra of NB (10^−7^ M) at 10 random points on a β-CD-SH-Ag film detected by droplet application, (**d**) Intensity of the 593 cm^−1^ peak at 10 random points on a β-CD-SH-Ag film detected by droplet application, (**e**) SERS spectra of NB (10^−7^ M) at 10 random points on a β-CD-SH-Ag film detected by soaking, (**f**) Intensity of the 593 cm^−1^ peak at 10 random points on a β-CD-SH-Ag film detected by soaking, (**g**) SERS spectra of NB (10^−7^ M) at 10 random points on a PVP-Ag film detected by droplet application, and (**h**) Intensity of the 593 cm^−1^ peak at 10 random points on a PVP-Ag film detected by droplet application.

The advantages of the method were explored by comparing it with some typical strategies. (1) Prepare silver film at the liquid interface without β-CD-SH (Figure 3a,b). Ten spectra of NB at 10^−7^ M were randomly collected from the silver film; the RSD of the Raman intensity at 593 cm^−1^ was as high as 54%, due to the nonuniformity of the NP distribution in the film. (2) Prepare blank β-CD-SH-assisted film and then drop analyte on it (Figure 3c,d). The RSD value was still up to 36%. (3) Prepare blank β-CD-SH-assisted film and immerse it in analyte solution for 20 min (Figure 3e,f). The results indicated sensitivity and uniformity (RSD = 19%) even worse than our method. Moreover, each sample required an additional 30–60 min for droplet application and drying, resulting in lower detection efficiency.

Additionally, the unique role of β-CD-SH in forming a monolayer film was explored by comparing it with other popular reagents. PVP-Ag film was prepared by using an oil/water/oil three-phase self-assembly process [37,38], followed by 10^−7^ M NB detection by both drop and immersion strategies. It was found that in the drop detection method, adding 10 μL of NB to the PVP-Ag film, led to the detachment of some Ag NPs from the silicon wafer, resulting in an RSD value of 45% (Figure 3g,h). Ag NP detachment from the film was much more significant during the immersion process due to the good hydrophilicity of PVP. Moreover, this method required pre-incubation of Ag NPs and the addition of a light oil phase after film formation to transfer the film to the upper layer, which significantly reduced detection efficiency. Secondly, by replacing the auxiliary reagent and using the same film-forming method, we found that when β-CD-SH was replaced with PFT, which can also regulate the spacing between Ag NPs, the sensitivity of SERS detection significantly decreased, with a detection limit of only 10^−8^ M (Figure S5). This may be due to PFT occupying the sites of Ag NPs, hindering contact between NB and Ag NPs. These results clearly indicate that β-CD-SH can efficiently capture target molecules, significantly enhancing the intensity and stability of detection signals. Specifically, β-CD-SH molecules achieve precise capture of NB molecules through their unique structural properties [39], optimizing the spatial arrangement of Ag NPs, and thereby improving detection sensitivity. The detection simplicity and efficiency are more advantageous compared with other popular self-assembly methods (Table S2).

### 3.3. Detection of Analytes with Different Solubility

**Figure 4:**
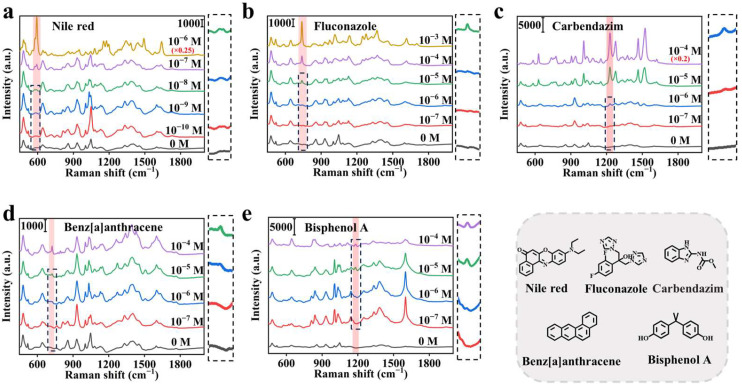
SERS spectra of (**a**) Nile red, (**b**) fluconazole, (**c**) carbendazim, (**d**) benz[a]anthracene, and (**e**) bisphenol A at different concentrations.

Detection generality of hydrophobic analytes was evaluated. In the detection of Nile Red (ring II breathing + β(C-CO-C) ring III at 592 cm^−1^) [40], the characteristic peaks could be identified at a concentration as low as 10^−9^ M (Figure 4a). For fluconazole, carbendazim, benzo[a]anthracene, and bisphenol A as typical hydrophobic organic pollutants with inherently low Raman activity, the detection limits could even reach 10^−6^ M with their characteristic peaks (Figure 4b–e) [41,42,43,44]. Specifically, in fluconazole, the 739 cm^−1^ characteristic peak corresponds to the vibration of the γ-CH group in the aromatic ring. In carbendazim, the characteristic peak at 1225 cm^−1^ is associated with the in-plane bending vibration of the N-H bond. In benzo[a]anthracene, the peak observed at 723 cm^−1^ is attributed to the ν_C-C_δ_C-C_ group. In bisphenol A, the characteristic peak at 1172 cm^−1^ reflects the vibration of para-substituted benzene.

For the detection of water-soluble molecules tested with crystal violet, malachite green, and rhodamine 6G, detection limits of 10^−6^ M, 10^−5^ M, and 10^−6^ M were obtained respectively from their corresponding characteristic peaks [40,45,46] (Figure S6a–c). The sensitivity is not as sound as that from the hydrophobic analyte, since water-soluble analytes have relatively weak binding with cyclodextrin and tend to freely distribute in the aqueous phase, lowering the possibility of their approaching the surface of Ag NPs. In contrast, hydrophobic analytes form more stable complexes with the β-CD cavity, allowing them to get closer to the nanoparticle surface, thereby achieving stronger signal enhancement.

### 3.4. Study on NB Release from Nanoplastics Under Different Conditions

Micro-/nanoplastics (MP/NPs) are emerging particulate pollutants in the water environment, and have a large specific surface area and strong hydrophobic surface. They can adsorb and concentrate organic compounds followed by a slow release process. The desorption properties are closely related to environmental conditions [47,48,49].

In this work, we investigated the effect of temperature, salinity, pH, and dissolved organic matter on the desorption behaviors of NB molecules from widespread PMMA nanoplastics in the environment. PMMA nanoplastics [50] of a 300 nm-diameter (Figure S7) were saturated with NB, and the PMMA-NB nanocomplex was incubated under varying environmental conditions. Then, the released NB was extracted via a simple liquid/liquid extraction method and detected via the proposed SERS detection method (Figure S2).

**Figure 5:**
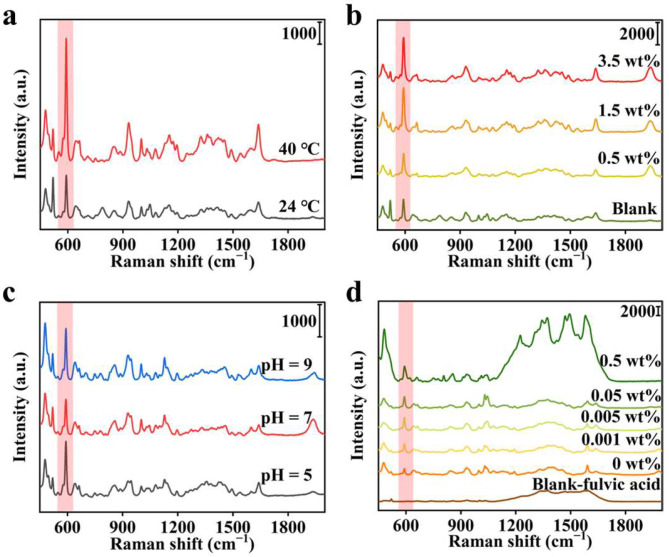
SERS spectra of NB released from PMMA under the influence of different (**a**) temperatures, (**b**) salinities, (**c**) pH values, and (**d**) concentrations of fulvic acid.

As illustrated in Figure 5a, temperature has a significant effect on the release of NB from nanoplastics. When the temperature increases from 24 °C to 40 °C, the release amount rises to 6.37 × 10^−7^ M, which is approximately 4.5 times higher than that at room temperature. This could be attributed to not only the enhanced thermal motion of NB molecules but also structural changes of binding sites on nanoplastics at elevated temperatures.

The influence of salinity on release behavior is also notable. As shown in Figure 5b, under low salinity conditions (0.5 wt%), there is virtually no change in the release amount. When salinity increases to 1.5 wt% and 3.5 wt%, the release amounts are nearly identical, and are twice those of the blank control. The increasing ionic strength alters the surface charge distribution of nanoplastics, the electrostatic effect, and interaction with NB molecules, and thus influences the adsorption/desorption equilibrium.

pH does not apparently induce NB release properties (Figure 5c). The release amounts at pH of 5, 7, and 9 are 1.17 × 10^−7^ M, 6.14 × 10^−8^ M, and 1.08 × 10^−7^ M, respectively. By contrast, the influence of co-existing dissolved organic matter is more significant (Figure 5d). When the concentration of fulvic acid is as low as 0.001 wt%, the release amount already increases by 1.7 times compared to the blank control, reaching approximately 1.56 × 10^−7^ M. At concentrations of 0.05 wt% and 0.5 wt%, the release amounts continuously increase to 2.25 × 10^−7^ and 3.45 × 10^−7^ M, respectively. This phenomenon can be attributed to competitive adsorption between fulvic acid molecules and NB molecules, where fulvic acid molecules occupy the adsorption sites on the nanoparticle surface, thereby promoting the release of NB.

## 4. Conclusions

This research presents an efficient self-assembly protocol for monolayer SERS detection mediated by β-CD-SH. The method enables the rapid formation of a compact Ag NP monolayer at the oil/water interface, leading to excellent reproducibility, with relative standard deviations of signal intensity less than 10% and low detection limits ranging from 10^−6^to 10^−9^ M for various hydrophobic analytes. Moreover, the method simplifies the detection process by integrating substrate assembly and analyte capture, eliminating the need for complex pretreatment and postmodification steps, thus enhancing detection efficiency compared to traditional methods. As a proof-of-concept environmental application, this method has been successfully employed to study the release behaviors of Nile blue as a hydrophobic pollutant adsorbed on PMMA nanoplastic surfaces. The findings reveal that temperature, salinity, and dissolved organic matter significantly influence the release process, providing crucial information for understanding the environmental fate of such pollutants. This work not only offers an effective SERS detection strategy but also holds extensive application prospects in environmental monitoring and chemical analysis.

## Data Availability

Detailed data can be obtained from the authors.

## Supplementary Materials

The following supporting information can be downloaded at https://www.mdpi.com/article/10.3390/bios15010052/s1: Figure S1. (a) Image of the monolayer film at the interface without β-CD-SH modification, (b) Image of the monolayer film at the interface with 10^−5^ M β-CD-SH modification; Figure S2. Plot of the logarithm of SERS peak intensity versus the logarithm of the concentration of NB at 593 cm^−1^; Figure S3. Raman spectra of the β-CD-SH reagent and β-CD-SH-Ag monolayer film; Figure S4. SERS detection spectra of NB (10^−7^ M) using monolayer films of β-CD-SH at various concentrations; Figure S5. SERS spectra of NB at different concentrations collected on PFT-Ag monolayer film; Figure S6. SERS spectra at different concentrations of (a) crystal violet, (b) malachite green, and (c) rhodamine 6G; Figure S7. SEM image of 300 nm PMMA nanoplastic; Table S1. Analysis of detection recovery rates for the established method; Table S2. Comparison of convenience in constructing SERS substrates via different self-assembly methods.
