# Supplementary material for: SERS Detection of Hydrophobic Molecules: Thio-β-Cyclodextrin-Driven Rapid Self-Assembly of Uniform Silver Nanoparticle Monolayers and Analyte Trapping
Source: Biosensors (Basel). 2025 Jan 15;15(1):52. doi: 10.3390/bios15010052 (PMC11763657; doi:10.3390/bios15010052)
Supplement: Supplementary file 1 [file biosensors-15-00052-s001.zip › biosensors-3402705-supplementary.pdf]

SERS Detection of Hydrophobic Molecules: Thio-β-Cyclodextrin-Driven Rapid Self-Assembly of Uniform Silver Nanoparticle Monolayers and Analyte Trapping

Qi Yuan [1,2] and Yunqing Wang [1,*]

[1] CAS Key Laboratory of Coastal Environmental Processes and Ecological Remediation, Yantai Institute of Coastal Zone Research, Chinese Academy of Sciences, Yantai 264003, China
[2] University of Chinese Academy of Sciences, Beijing 100049, China
* Correspondence: yqwang@yic.ac.cn

Supplementary Materials

Experimental
Synthesis of Citrate-Coated Ag Nanoparticles
Self-Assembly of Pvp-Capped Ag Nanoparticle Film
Synthesis of PMMA NPs
Figures
Figure S1. (a) Image of the monolayer film at the interface without β-CD-SH modification, (b) Image of the monolayer film at the interface with 10^{-5} M β-CD-SH modification.
Figure S2. Plot of the logarithm of SERS peak intensity versus the logarithm of concentration of NB at 593 cm^{-1}.
Figure S3. Raman spectra of the β-CD-SH reagent and β-CD-SH-Ag monolayer film.
Figure S4. SERS detection spectra of NB (10^{-7} M) using monolayer films of β-CD-SH at various concentrations.
Figure S5. SERS spectra of NB at different concentrations collected on PFT-Ag monolayer film.
Figure S6. SERS spectra at different concentrations of (a) crystal violet, (b) malachite green, and (c) rhodamine 6G.
Figure S7. SEM image of 300 nm PMMA nanoplastic.
Tables
Table S1. Detection recovery rates for the established method.
Table S2. Comparison of convenience in constructing SERS substrates via different self-assembly methods.

Experimental

Synthesis of Citrate-coated Ag Nanoparticles

Ag nanoparticles were synthesized via the Lee–Meisel method [1]. Initially, 22.5 mg of $AgNO_3$ was dissolved in 125 mL of ultrapure water in a three-necked round-bottom flask fitted with a reflux condenser. The solution was then heated until boiling, followed by the addition of 2.5 mL of a 1 wt% sodium citrate solution. The boiling conditions were maintained under vigorous mechanical stirring for 0.5 h, after which the mixture was allowed to cool to room

temperature, then stored at 4 °C for subsequent use.

Self-assembly of PVP-capped Ag Nanoparticle Film

Following the method of Au monolayer self-assembly of Chen et al., with adjustments and optimization, a Ag monolayer film was prepared using an oil/water/oil three-phase self-assembly process based on the Marangoni effect [2]. Initially, 2 mL of a PVP/ethanol solution (1 wt%) was mixed with 2 mL of citrate-capped Ag NPs and sonicated for 30 min to replace the hydrophilic ligand CTA^+ on the Ag NPs with PVP. The resulting PVP-protected Ag NPs were then centrifuged at 9000 rpm for 10 min, and the supernatant was removed. The precipitate was redispersed in 0.15 mL of ethanol.

The PVP-coated Ag NPs were then mixed with 1 mL of dichloromethane, followed by the addition of 1.8 mL of deionized water. After vigorously shaking for 1 min, a thin metal film formed at the dichloromethane/water interface. Subsequently, 400 μL of hexane was carefully added along the wall of the centrifuge tube. The nanoparticles were transported to the hexane/water interface, forming a dense, mirror-like film. Finally, the upper layer of hexane was removed, and the Ag film was transferred onto a silicon wafer to dry, yielding a PVP-capped Ag film for further experiments.

Synthesis of PMMA NPs

PMMA nanoplastics (300 nm in size) were synthesized through a method [3] involving mechanical stirring and polymerization under a nitrogen atmosphere. Initially, 120 mL of water was introduced into a 250 mL round-bottom flask, which was then subjected to mechanical stirring at 300 rpm for 5 min. Following this, 10 g of MMA was added to the flask, and the stirring was continued for an additional 10 min. The flask was subsequently immersed in a water bath, and the stirring was maintained for 30 min once the bath temperature reached 75 ° C. At this point, 0.1 g of KPS was introduced to initiate polymerization. The polymerization reaction was allowed to proceed for 5 h. Ultimately, PMMA nanoplastics with a diameter of approximately 300 nm were obtained.

Figures

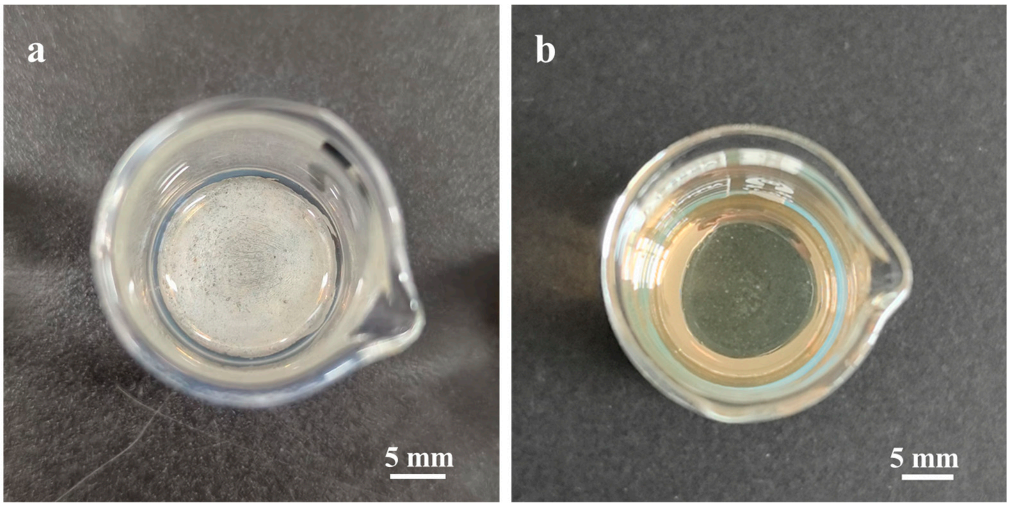

Figure S1. (a) Image of the monolayer film at the interface without β-CD-SH modification, (b) Image of the monolayer film at the interface with 10^{-5} M β-CD-SH modification.

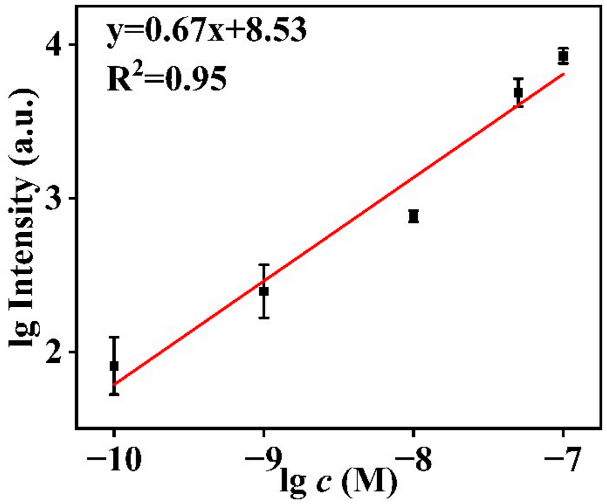

Figure S2. Plot of the logarithm of SERS peak intensity versus the logarithm of the concentration of NB at 593 cm^{-1}. Error was unweighted in the fitting.

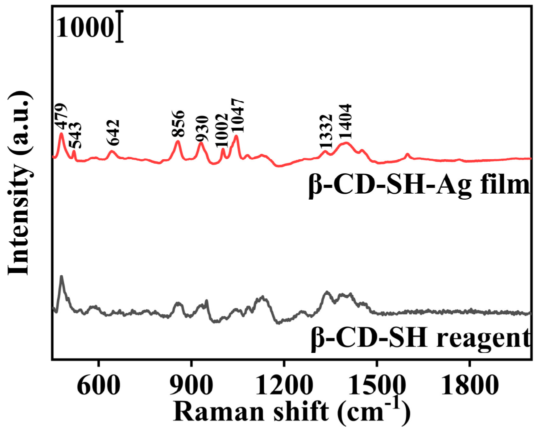

Figure S3. Raman spectra of the β-CD-SH reagent and β-CD-SH-Ag monolayer film.

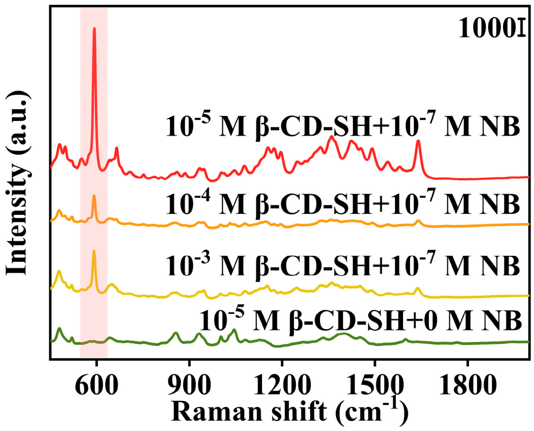

Figure S4. SERS detection spectra of NB (10^{-7} M) using monolayer films of β-CD-SH at various concentrations.

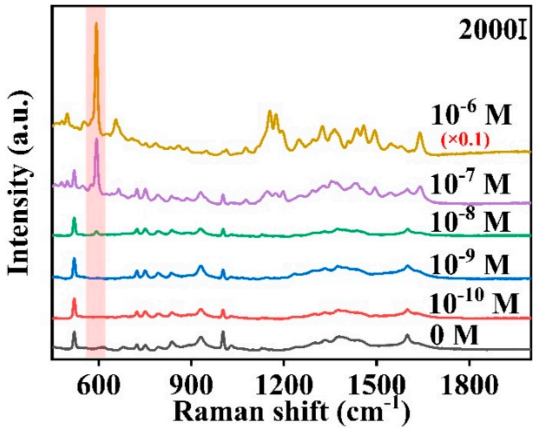

Figure S5. SERS spectra of NB at different concentrations collected on PFT-Ag monolayer film.

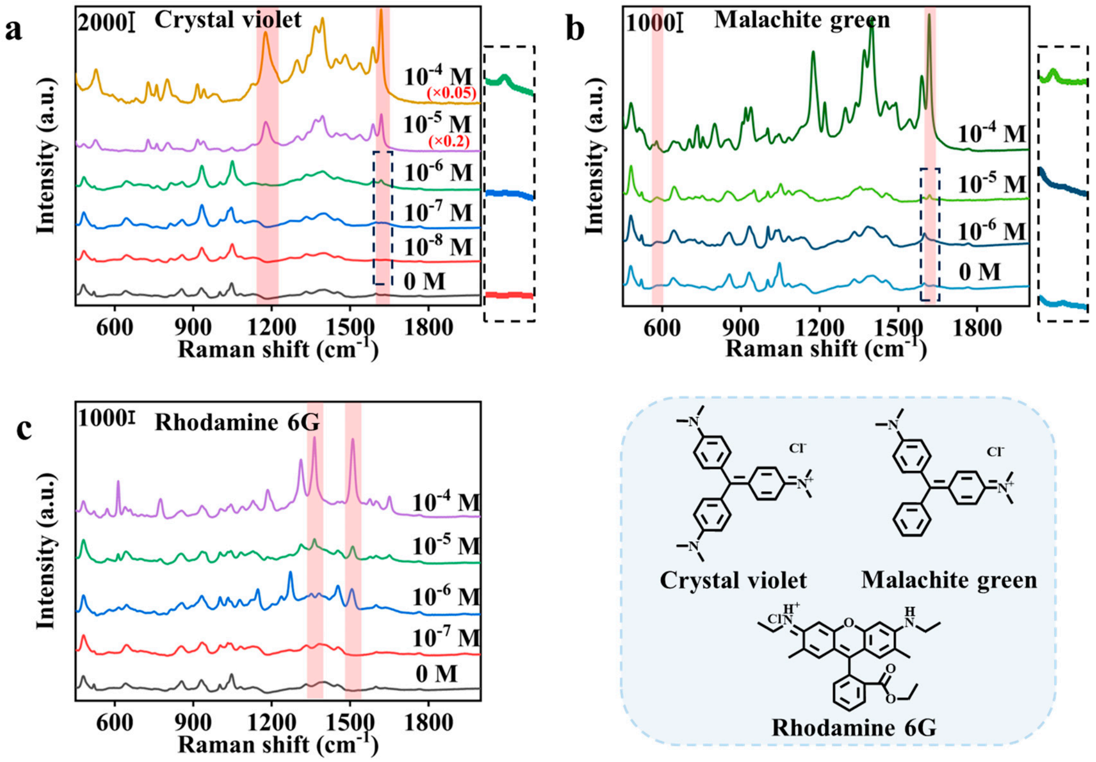

Figure S6. SERS spectra at different concentrations of (a) crystal violet, (b) malachite green, and (c) rhodamine 6G.

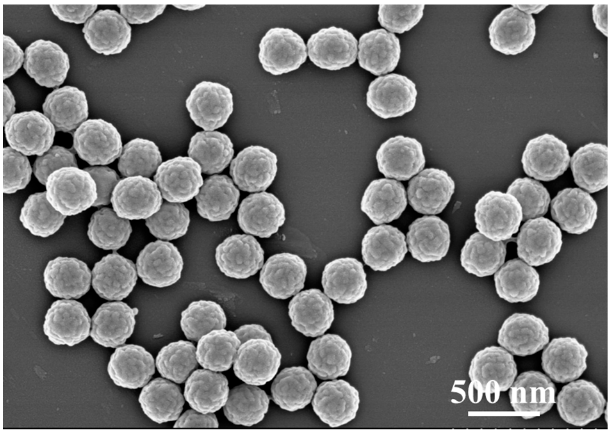

Figure S7. SEM image of 300 nm PMMA nanoplastic.

Tables

Table S1. Analysis of detection recovery rates for the established method.

Labeling concentration	Intensity value of feature peak	Quantitative method	Quantified concentration (Converted)	Recovery rate
2×10^{-8} M NB (2 mL, dissolved in organic phase)	1944.96	Y = 0.67x+8.53	$1.50*10^{-8}$ M	75 %

Table S2. Comparison of convenience in constructing SERS substrates via different self-assembly methods.

SERS substrates	Nanoparticle preincubation	Assembly method and duration	Modification after assembling	Dot blot or immersion capture of target molecules	Ref.
Au nanosphere monolayers	yes	Gold nanosphere monolayers were self-assembled via ligand exchange, solvent phase separation, and interfacial deposition on hydrophilic substrates.	yes	yes	[2]
oCDs/Ag NPs@glass SERS sensor	no	The glass slide was placed into the mixture solution of NaOH, CPC, and $AgNO_3$ for incubation. **12 h**	no	yes	[4]
β-CD functionalized silver nanoparticle monolayer	no	The centrifuged and redispersed 100 nm Ag NPs in methanol were mixed with toluene in a glass beaker, followed by immediate addition of a specified volume of MQ water. **1 h**	yes	yes	[5]

Au/Ag self-assembled monolayers	no	Au/Ag SAMs were fabricated by dissolving nanospheres in a mixed solution, evaporating carrier solvents, and generating nanofilms at the air/solution interface. **12 h**	no	yes	[6]
This work	no	Ag nanoparticles and organic phase (containing analytes and β-CD-SH) were mixed and self-assembled into a monolayer at the oil/water interface.**40 s**	no	no	

References

1. Lee, P. C.; Meisel, D. Adsorption and surface-enhanced Raman of dyes on silver and gold sols. J. Phys. Chem. 1982, 86 (17), 3391-3395.
2. Chen, X. Y.; Cui, A. R.; He, M. Y.; Yan, M.; Zhang, X. C.; Ruan, J.; Yang, S. K. Slippery Au Nanosphere Monolayers with Analyte Enrichment and SERS Enhancement Functions. Nano Lett. 2023, 23 (14), 6736-6743.
3. Tang, B.; Wu, C.; Lin, T.; Zhang, S. Heat-resistant PMMA photonic crystal films with bright structural color. Dyes and Pigments 2013, 99 (3), 1022-1028.
4. Qiu, J. Y.; Chu, Y. J.; He, Q. H.; Han, Y. K.; Zhang, Y.; Han, L. A self-assembly hydrophobic oCDs/Ag nanoparticles SERS sensor for ultrasensitive melamine detection in milk. Food Chem. 2023, 402, 134241.
5. Liu, J.; Zhang, C. C.; Zhang, S. X.; Yu, H. J.; Xie, W. A versatile β-cyclodextrin functionalized silver nanoparticle monolayer for capture of methyl orange from complex wastewater. Chin. Chem. Lett. 2020, 31 (2), 539-542.
6. Tian, Y.; Zhang, H.; Xu, L. L.; Chen, M.; Chen, F. Self-assembled monolayers of bimetallic Au/Ag nanospheres with superior surface-enhanced Raman scattering activity for ultra-sensitive triphenylmethane dyes detection. Opt. Lett. 2018, 43 (4), 635-638.
